# Top‐down control by an aquatic invertebrate predator increases with temperature but does not depend on individual behavioral type

**DOI:** 10.1002/ece3.4367

**Published:** 2018-07-22

**Authors:** Travis Ingram, Zuri D. Burns

**Affiliations:** ^1^ Department of Zoology University of Otago Dunedin New Zealand

**Keywords:** activity, animal personality, backswimmer, Notonectidae, reaction norm, trophic cascade

## Abstract

Variation in behavioral traits among individuals within a population can have implications for food webs and ecosystems. Temperature change also alters food web structure and function, but potential interactions between warming and intraspecific behavioral variation are largely unexplored. We aimed to test how increased temperature, individual activity level of a predatory backswimmer (*Anisops assimilis*), and their interaction influenced the strength of top‐down control of zooplankton and phytoplankton. We used stable isotopes to support our assumption that the study population of *A. assimilis* is zooplanktivorous, and behavioral trials to confirm that activity level is a repeatable trait. We established freshwater microcosms to test for effects of warming, backswimmer presence, and backswimmer behavioral type on zooplankton density, zooplankton composition, and phytoplankton chlorophyll *a*. Top‐down control was present and was generally stronger at increased temperature. There was no indication that predator behavioral type influenced the strength of top‐down control either on its own or interactively with temperature. Predator behavioral type may not be associated with ecologically important function in this species at the temporal and spatial scales addressed in this study, but the links between behavior, temperature, and food web processes are worthy of broader exploration.

## 1 INTRODUCTION

Individuals within populations vary in phenotypic traits including behavior and resource use, and there are indications that this variation can have important consequences for populations, communities, and ecosystems (Bolnick et al., 2011). Numerous studies have found divergent effects of distinct genotypes or populations on ecosystem processes (Des Roches et al., 2018; Harmon et al., 2009; Hughes, Inouye, Johnson, Underwood, & Vellend, 2008), although manipulations of the variance in continuously distributed traits are rare (Carlson & Langkilde, 2017; Ingram, Stutz, & Bolnick, 2011). Environmental drivers such as temperature change can directly impact food webs, but can also influence the expression of individual variation in phenotypes including behavior. Therefore, we can predict that changes in the environment could influence food web and ecosystem processes both directly and indirectly via effects on phenotypic variation.

Activity level is a repeatable behavioral trait in many species, and is often correlated with other traits such as boldness and aggression to form behavioral syndromes or “animal personalities” (Bell, Hankison, & Laskowski, 2009; Sih, Bell, & Johnson, 2004; Sih, Cote, Evans, Fogarty, & Pruitt, 2012). “Proactive” behavioral syndromes associated with high activity levels can also be linked to metabolic rates and life history strategies (Holtmann, Lagisz, & Nakagawa, 2017; Réale et al., 2010). Because activity level can influence the extent to which an individual will encounter different prey types, predators, or microhabitats, we can anticipate that active and inactive individuals will differ in their ecological role as well (Michalko & Pekar, 2017; Royauté & Pruitt, 2015; Toscano & Griffen, 2014). For example, in larvae of the dragonfly *Epitheca canis*, active individuals consume more zooplankton while decreasing the relative abundance of the most efficient grazer, the cladoceran *Daphnia catawba* (Start & Gilbert, 2017). This results in the strength of trophic cascades increasing with individual activity level, suggesting ecosystem‐scale consequences of behavioral variation.

A largely isolated line of research has recently investigated the extent to which top‐down control varies with temperature, usually with the aim of assessing how climate warming is likely to impact food web structure and function (O'Connor, Gilbert, & Brown, 2011; Shurin, Clasen, Greig, Kratina, & Thompson, 2012). While the particulars vary from system to system, in many cases increased temperature results in stronger top‐down control and higher heterotroph:autotroph ratios (Barton, Beckerman, & Schmitz, 2009; O'Connor, Piehler, Leech, Anton, & Bruno, 2009; Shurin et al., 2012). This result is consistent with theoretical predictions that incorporate the greater temperature dependence of respiratory metabolism relative to photosynthesis (O'Connor et al., 2011). This temperature dependence has important implications for how both pairwise trophic interactions and broader functioning of food webs are affected by changes in climate.

Despite the growing evidence that both intraspecific behavioral variation and changes in temperature can influence the strength of food web processes, little if any consideration has been given to the potential interaction between these phenomena. Behaviors such as activity level are often temperature‐sensitive, particularly in ectotherm predators (Biro, Beckmann, & Stamps, 2010; Briffa, Bridger, & Biro, 2013; Huey & Kingsolver, 1989; Pruitt, Demes, & Dittrich‐Reed, 2011). An increase in temperature often increases the mean activity level, and may alter the degree of variation in behavior within a population (Nakayama, Laskowski, Klefoth, & Arlinghaus, 2016). The thermal reaction norm—the change in behavior with a change in temperature—may be consistent among individuals (Pruitt et al., 2011), or individuals may differ in the magnitude or even direction of their response to temperature (Biro, O'Connor, Pedini, & Gribben, 2013). Temperature therefore has the potential to influence food web processes either directly or via changes in the expression of individual behavior, but to our knowledge, these links have not been tested.

Here, we use a freshwater microcosm experiment to test whether the magnitude of top‐down control of zooplankton and phytoplankton depends on the behavioral type of an aquatic invertebrate predator (the New Zealand backswimmer *Anisops assimilis*), temperature, or their interaction. We predicted first that backswimmer presence would result in top‐down control (decreased zooplankton density and increased phytoplankton density), and that more active individuals would exert stronger top‐down control by consuming more zooplankton, especially daphniid cladocerans. We also predicted that the strength of top‐down control would be enhanced at higher temperatures. At last, we predicted that a moderate degree of daytime warming could either enhance or decrease the strength of the relationship between activity and top‐down control.

## 2 METHODS

### 2.1 Study site

We collected all organisms used in this study from a single natural pond from which backswimmers and zooplankton could easily be collected, as most other nearby fishless ponds containing backswimmers were artificially dug for agriculture or fire fighting. Our sampling was conducted at “Lake Whare,” a small (0.03 ha) pond on the slope of Swampy Mountain in Dunedin, New Zealand (45.809°S, 170.457°E). This is a shallow (1–1.5 m) but permanent pond surrounded by regenerating scrub vegetation, with a margin consisting of partly submerged grasses. The pond is at an elevation of ~330 m, and has been observed to have ice formation around the margins in the winter but is unlikely to freeze over for any extended period. The most common macroinvertebrate at the time of sampling was the backswimmer *A. assimilis*, one of two widespread notonectid species present in New Zealand (Young, 2010). The water boatman *Sigara arguta* was also common and the larval damselfly *Austrolestes colensonis* was present at lower density.

### 2.2 Stable isotope analysis

To confirm that zooplankton were likely the primary food source for backswimmers at the time and site of sampling, on 6 July 2017 we collected backswimmers (*n* = 30) using sweep nets. Backswimmers were euthanized by freezing, then examined under a dissecting microscope to confirm species identity, determine sex, and measure total body length. We also collected bulk samples of zooplankton (*n* = 5), *S. arguta* (*n* = 10) and *A. colensonis* larvae (*n* = 4).

We froze individual macroinvertebrates and samples of bulk zooplankton, then dried them at 60°C for 48 hrs. We homogenized each sample with a mortar and pestle and packaged ~0.8 mg into a tin capsule for stable isotope analysis. Stable isotope analysis of carbon and nitrogen was carried out on a Europa Hydra stable isotope mass spectrometer (Europa Scientific) interfaced to a Carlo Erba elemental analyser (NC2500; Carlo Erba) at the Iso‐trace laboratory (Department of Chemistry, University of Otago). Precision based on internal replicates was ±0.3 ‰ for δ^15^N and ±0.2 ‰ for δ^13^C. Isotope values are expressed in the standard delta notation relative to the international standards Pee Dee belemnite limestone carbon and atmospheric nitrogen. We applied a standard arithmetic formula to calculate δ^13^C_norm_, accounting for the effect of lipids in depleting ^13^C relative to the diets of aquatic organisms (Post et al., 2007).

We did not have multiple likely prey items to assess the relative contribution of zooplankton, as *S. arguta* and *A. colensonis* are too large to be prey to *A. assimilis* under most circumstances, and other potential prey such as dipteran larvae and pupae were not found in our sampling. Instead, we qualitatively examined whether the position of *A. assimilis* on a δ^15^N‐δ^13^C biplot was consistent with a primary diet of zooplankton (i.e., a higher δ^15^N and a broadly similar δ^13^C compared to bulk zooplankton). We also tested for intraspecific variation in diet associated with sex or body length, to assess whether these factors could confound statistical analysis of behaviorally mediated top‐down control. We fit separate linear models with δ^15^N or δ^13^C_norm_ as the response variable and sex, body length, and their interaction as predictors. To facilitate interpretation of coefficients, body length was first scaled by subtracting the mean and dividing by two standard deviations (Gelman, 2008). All analyses were carried out in the R environment (R Core Team 2018).

### 2.3 Behavioral assay and repeatability

We assayed the repeatability of individual backswimmer behavior using open field activity tests at two temperatures, 10 and 18°C (see “Microcosm experiment *”* for explanation). Backswimmers were collected on August 11, 2017 using a metal sieve attached to a pole, then held in pond water for <2 hrs before transfer to the University of Otago. We excluded any larval instar stages lacking wings, as well as individuals with visibly heavy loads of ectoparasitic hydracarine mites, although a few individuals were found to have one or a few mites when examined under a microscope later (*Hydrachna* sp.; Marples, 1962). Backswimmers were individually transferred to 250 ml plastic containers filled with 200 ml of natural spring water “Speight's water”, then 23 individuals were placed in a room maintained at a constant temperature of 10°C and 20 were placed in a room maintained at 18°C. They were given 2 days to acclimate to the laboratory without feeding, then their activity level was assayed in the same room (at either 10 or 18°C).

For each assay, an individual backswimmer was gently placed in a white square plastic container (diameter 17 cm, depth 8.5 cm) filled to a depth of 1 cm with spring water. Videos were recorded for 360 s using a GoPro Hero3 camera (GoPro Inc., San Mateo, CA, USA) positioned 50 cm directly above the arena. Sheets of white polystyrene were placed around the arenas and recordings were started and stopped via remote control to limit external stimuli. The backswimmer was returned to its holding container following the trial. Later on the same day, after a period of at least 3 hrs, each backswimmer was tested again at the same temperature using an identical setup. Backswimmers were then transferred to the other room (i.e., from 10 to 18°C or vice versa) and allowed to acclimate for a further 2 days, again without feeding. Two trials were conducted at the new temperature, again separated by a minimum of 3 hrs, then backswimmers were euthanized by freezing and later examined to determine body length, sex, and the presence of any ectoparasitic mites.

We used the software EthoVision XT11 (Noldus Information Technology Inc., Wageningen, The Netherlands; Noldus, Spink, & Tegelenbosch, 2001) to track the path moved by the backswimmer during each trial. We disregarded the first 60 s after transfer to the container, treating this as a brief additional acclimation period (backswimmers had already had 2 days to acclimate to the water and temperature of the trial conditions). We extracted the total distance moved during the remaining 300 s trial period, then calculated the average velocity in cm/s as a measure of activity. We thus obtained four measures of activity, two each at 10 and 18°C, for each of the 43 individuals.

We first estimated variation among individuals separately at 10°C and at 18°C by fitting mixed effects models with the R package “lme4,” with individual identity as a random effect and order (i.e., whether individuals were first assayed at 10 or 18°C) included as a fixed effect. We then combined all data and fit a model with temperature as a fixed effect to estimate the average reaction norm for activity level, in addition to effects of individual, order, and a temperature × order interaction. In all cases there was no significant main or interactive effect of order, so we removed it from the model to simplify interpretation. We also found no effect of sex, body length, or ectoparasite presence on activity, so we do not consider these factors further. For each of these models, we also used the “rptGaussian” function in the R package rptR (Stoffel, Nakagawa, & Schielzeth, 2017) to estimate repeatability as the proportion of variance explained by individual identity. We then tested for variation in the reaction norm among individuals by fitting a random slopes model where individuals can differ in their response to temperature. We compared this to the previous random intercept model using a likelihood ratio test.

### 2.4 Microcosm experiment

We established a microcosm experiment with six treatments: two daytime temperature treatments (10 or 18°C) crossed with three food web treatments named for the top trophic level present: phytoplankton (P), zooplankton (Z), and backswimmer (B). The design was deliberately unbalanced, with each temperature treatment having 10 replicates each of the phytoplankton and zooplankton treatments and 30 replicates of the backswimmer treatment, to allow power to detect effects of behavioral type within the backswimmer replicates (Start & Gilbert, 2017). We established 100 indoor microcosms in 17 × 17 × 8 cm square white plastic containers, 50 in each of two temperature‐controlled rooms in the Parker Animal Facility at the Department of Zoology at the University of Otago, New Zealand. Microcosms were placed on the top shelves of five shelf racks arranged around the edge of each room, with treatments assigned in a stratified random manner with two phytoplankton, two zooplankton, and six backswimmer replicates randomly placed on each shelf. Both rooms were set to a regime of 11 hrs “daytime” (with lighting consisted of 36 W “cool white” fluorescent ceiling lamps approximately 2 m above the microcosms) and 10 hrs “nighttime,” with a 1.5 h transitional period mimicking dawn and dusk. Both treatments were maintained with a nighttime temperature of 8°C, and target daytime temperatures of 10 and 18°C, meant to approximate ambient conditions and a prolonged heat wave in the austral winter. A HOBO pendant data logger (Onset, Bourne, MA) was placed in a spare microcosm container in each room throughout the experiment (September 2 to September 16, 2017), and an additional logger was deployed 10 cm below the surface of Lake Whare from 6 to 29 September.

Backswimmers, zooplankton and phytoplankton were collected from Lake Whare 3 days before the start of the experimental period. Adult backswimmers lacking visible ectoparasites were kept in pond water for <2 hrs, then returned to the University of Otago and then held overnight at 8°C. Bulk zooplankton was collected using a pole net swept repeatedly through the water just below the surface, filtered through a 250 μm plankton sieve, and aggregated into a container with pond water. 80 L of pond water was poured through the 250 μm sieve and collected in buckets for use as a source of phytoplankton as well as nutrients, bacteria and other biotic and abiotic components of the pond water. Five 10 L samples were filtered to estimate zooplankton density in the pond, and three water samples were taken to infer phytoplankton density.

Each microcosm was allocated 0.75 L filtered pond water, 0.8 L spring water, and an equal aliquot of a culture of *Cryptomonas* sp. This ensured that all treatments received a algal base of primary production, although we note that in addition to natural algal communities and *Cryptomonas* cultures, all microcosms received protists, bacteria, and potentially zooplankton resting stages or instars <250 μm. Each microcosm received an aliquot of nutrient broth containing 4.0 mg NaNO_3_ and 0.32 mg NaH_2_PO_4_ to ensure an adequate supply for phytoplankton growth. Zooplankton was added to the appropriate microcosms the day after algae and water were set up. A large quantity of bulk zooplankton was split repeatedly with a Folsom plankton splitter, to obtain 80 approximately equal subsamples of zooplankton from the pond. The zooplankton subsamples were added to the microcosms with 50 ml of spring water, and 50 ml of spring water was added to the phytoplankton treatments, bringing the volume to 1.6 L in each microcosm.

On the same day zooplankton were added, each of the 60 backswimmers had its activity level assayed once as described above, at the daytime temperature of 10°C in the colder room. To ensure that each temperature treatment received a comparable distribution of behavioral types, we sorted the backswimmers by activity level, divided them into deciles of six, and then randomly assigned three from each decile to each temperature treatment. The day following the behavioral assay, each backswimmer was placed in the appropriate room in its 250 ml container to allow gradual acclimation to temperature change for the 18°C treatment, then gently transferred to its assigned microcosm.

The experiment was run for 14 days. After the first 7 days, the temperature regimes and microcosm containers were swapped between rooms to ensure that any room effects were not wholly confounded with temperature treatment. The transfer was made early in the morning to minimize any disruption to the temperature treatments. Twice weekly, containers were topped up to 1.6 L with spring water to compensate for evaporative loss (particularly in the 18°C treatment). At the end of the experiment, backswimmers were removed, euthanized by freezing, and preserved in ethanol. All water in the container was poured through a 250 μm plankton sieve to sample zooplankton. The top 1.2 L of filtered water was taken for phytoplankton chlorophyll analysis, with care taken to avoid including any attached or settled algae.

Each phytoplankton sample was transferred to a glass filter paper (Advantec GF‐50, 47 mm; Toyo Roshi Kaisha Ltd., Tokyo, Japan) using vacuum filtration, wrapped in foil and stored at −20°C. Chlorophyll was extracted in 90% ethanol following Biggs and Kilroy (2000). Chl *a* concentration was then determined spectophotometrically using a FLUOstar Omega multidetection microplate reader (BMG Labtech GmbH, Offenburg, Germany), with readings corrected for phaeopigments then converted to μg/L chl *a*. Zooplankton samples were preserved in ethanol and later counted under a dissecting microscope. We identified zooplankton to the taxonomic groups calanoid copepods (*Boeckella* sp.), cyclopoid copepods (*Cyclops* sp.), daphniid cladocerans (*Ceriodaphnia dubia*), chydorid cladocerans (*Chydorus* sp.), and ostracods, and calculated total zooplankton density. We also calculated the proportion of daphniids in each sample, as daphniid cladocerans may be both preferred by notonectids and more likely to control phytoplankton growth (Arner, Koivisto, Norberg, & Kautsky, 1998; Start & Gilbert, 2017). Backswimmers were examined under a dissecting microscope to measure body length, determine sex, and confirm that all individuals were adult *A. assimilis* (Young, 2010).

We subdivided the data into sets appropriate to testing hypotheses about top‐down control by zooplankton (food web treatments P + Z), top‐down control by backswimmers (treatments Z + B), and effects of behavior type (B only). We used a two‐way ANOVA with data from the P + Z treatments to test for effects of zooplankton addition, temperature, and their interaction on phytoplankton chl *a*. We then used two‐way ANOVAs with data from the Z + B treatments to test for effects of backswimmer addition, temperature, and their interaction on total zooplankton density (log‐transformed for normality), proportion daphniids (logit‐transformed for normality), and phytoplankton chl *a*.

We used linear models with data from only the B treatments to test for effects of backswimmer behavior on zooplankton density, proportion daphniids, and phytoplankton chl *a*. We included as predictors temperature, backswimmer activity, and a temperature × activity interaction, with backswimmer sex and total length included as covariates. Activity and total length were scaled by subtracting the mean and dividing by two standard deviations (Gelman, 2008).

## 3 RESULTS

### 3.1 Stable isotope analysis

**Figure 1:**
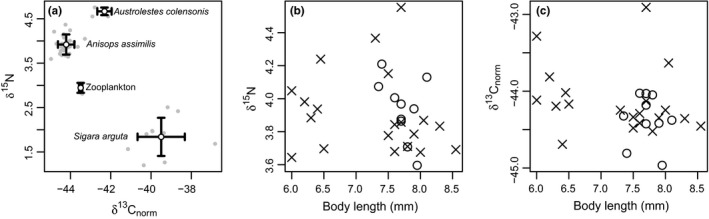
Stable isotope data from *Anisops assimilis* and other invertebrates in Lake Whare. (a) Mean (±1 *SD*) δ^15^N and lipid‐normalized δ^13^C for the three common macroinvertebrate species present in the pond as well as bulk zooplankton. (b) δ^15^N and (c) δ^13^C_norm_ compared to body length for male (×) and female (o) *A. assimilis* (note the clear size gap between larval instars <7 mm and adults >7 mm; all seven larval instars in this sample happened to be male)

*Anisops assimilis* was somewhat enriched in δ^15^N and depleted in δ^13^C_norm_ compared to bulk zooplankton samples. The larval damselfly *A. colensonis* was somewhat enriched in both δ^15^N and δ^13^C_norm_ compared to *A. assimilis*, while the water boatman *S. arguta* was substantially depleted in δ^15^N and enriched in δ^13^C_norm_ (Figure 1a). These observations are generally consistent with diet studies of the macroinvertebrate taxa concerned: *A. colensonis* has been observed to feed on multiple macroinvertebrate taxa including *Anisops* sp. in addition to zooplankton (Crumpton, 1979), while New Zealand corixids such as *S. arguta* are thought to be primarily detritivorous (Young, 2010).

Backswimmer δ^15^N was not affected by sex (β_male_ = −0.14 ± 0.14 standard error, *p* = 0.33), scaled total length (β = −0.97 ± 0.94, *p* = 0.32), or their interaction (β = 0.84 ± 0.96, *p* = 0.39; Figure 1b). Likewise, backswimmer δ^13^C_norm_ was unrelated to sex (β_male_ = 0.18 ± 0.24, *p* = 0.46), scaled total length (β = −0.29 ± 1.65) or their interaction (β = −0.02 ± 1.68, *p* = 0.99; Figure 1c).

### 3.2 Behavioral assay and repeatability

**Figure 2:**
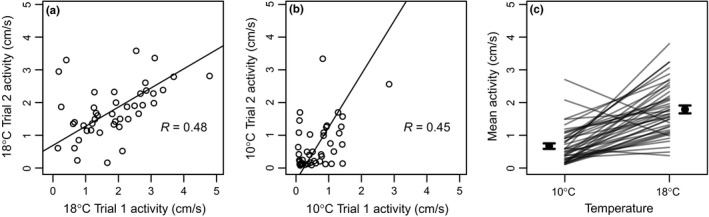
Repeatability of activity levels within and between temperatures. When individuals are assayed (a) twice at 18°C or (b) twice at 10°C, activity (measured as mean velocity in an open field test) was moderately repeatable (major axis regression lines are shown for visual effect). (c) Combining assays at both temperatures reveals an increase in activity at higher temperature as well as heterogeneous reaction norms among individuals (endpoints of each line represent the mean of two trials for the same individual; error bars are mean ± 1 *SE*)

We found that individual activity level was repeatable when assayed at the same temperature. When the two activity measures per individual at 18°C were analyzed together they had a repeatability of *R* = 0.478 (95% CI 0.231–0.677, *p* = 0.0005; Figure 2a), while the two measures at 10°C had a repeatability of *R* = 0.453 (95% CI = 0.191–0.672, *p* = 0.001; Figure 2b). When temperature was incorporated into the mixed effects model, there was a clear increase in activity level between 10 and 18°C (difference 1.17 cm/s, 95% profile confidence interval 0.88–1.46 cm/s). Neither order of treatments nor an order × temperature interaction had any effect (confidence intervals clearly overlapped zero), so these terms were not included in the final model. With temperature included, the repeatability decreased to *R* = 0.231 (95% CI = 0.067–0.386, *p* = 0.0005). The random slopes model revealed considerable heterogeneity in individuals’ change in activity with temperature (Figure 2c), and was strongly supported over a random intercept model where individuals were assumed to respond similarly to temperature (χ^2^ = 15.5, *df* = 2, *p* = 0.00044).

### 3.3 Microcosm experiment

Our temperature manipulation successfully mimicked the day–night cycles in Lake Whare, with water warming through the day toward the target daytime temperature of 10°C or 18°C, then dropping rapidly to the nighttime temperature of 8°C (most of the temperature change happened between 6–10 a.m. and 6–10 p.m.). The 10°C treatment corresponded well to the mean daily maximum temperature recorded in the pond of 11°C (*SD* 2.72°C), and the nighttime temperature of 8°C matched the mean daily minimum of 8.1°C (*SD* 1.5°C) in the pond. The 18°C treatment was close to the maximum temperature recorded in the pond of 17.2°C.

Two backswimmers died during the experiment, while another two migrated out of their microcosms and were found dead later. For two of these that occurred in the first 3 days of the experiment (one in each temperature treatment), we replaced the missing backswimmer with another individual whose behavior had been assayed at the same time as the initial group; the other two microcosms (also one per temperature treatment) were left empty and excluded from all analyses.

Zooplankton communities in the microcosms consisted of *Boeckella* sp. (79.7%), *Cyclops* sp. (7.5%), *Ceriodaphnia dubia* (6.8%), *Chydorus* sp. (4.6%) and ostracods (<0.1%). Copepods and *C. dubia* were generally of a comparable size range (most individuals 0.8–1.2 mm in length), while *Chydorus* sp. and ostracods were roughly half this size. Zooplankton presence and warming interactively decreased phytoplankton chl *a* in the microcosms (2‐way ANOVA: Temperature *F*_1,36_ = 14.2, *p* = 0.0006; Zooplankton *F*_1,36_ = 47.0, *p* < 0.0001; Interaction *F*_1,36_ = 12.0, *p* = 0.001). Zooplankton presence reduced phytoplankton chl *a* from an average of 6.0 ± 0.89 (standard error) to 1.9 ± 0.29 μg/L at 10°C, and from 14.8 ± 2.21 to 2.3 ± 0.29 μg/L at 18°C (for reference the average chl *a* in water from Lake Whare was 1.2 ± 0.25 μg/L).

**Figure 3:**
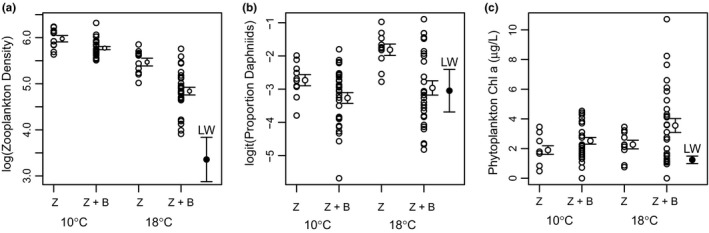
Top‐down control of (a) zooplankton density, (b) proportion of daphniid zooplankton, and (c) phytoplankton chl *a* by *A. assimilis*. Symbols show individual data points and error bars show mean ± 1 *SE* for zooplankton (Z) and backswimmer addition (Z + B) treatments at 10 and 18°C. In each panel, the error bar below the text “LW” indicates the mean equivalent value (±1 *SE*) measured in Lake Whare at the time of sampling (*n* = 5 zooplankton and 3 phytoplankton samples)

Backswimmer addition and warming interactively impacted zooplankton density, and independently affected zooplankton composition and chl *a* (Figure 3). Total zooplankton density (log‐transformed) was negatively affected by temperature (*F*_1,74_ = 126.8, *p* < 0.0001), by backswimmer addition (*F*_1,74_ = 24.7, *p* < 0.0001), and by a synergistic interaction (*F*_1,74_ = 6.5, *p* = 0.013). Backswimmer presence reduced zooplankton density from an average of 402.6 ± 27.0 to 327.2 ± 12.2 individuals per microcosm at 10°C (implying an average consumption rate of 5.5 individuals per backswimmer per day), and from 244.9 ± 19.8 to 139.1 ± 11.6 at 18°C (7.5 individuals per backswimmer per day). Zooplankton densities at the conclusion of the experiment were still higher than in the pond at the time of sampling (45.1 ± 22.2 individuals per equivalent volume; Figure 3a). The proportion of daphniids was increased by temperature (*F*_1,74_ = 4.7, *p* = 0.032) and decreased by backswimmer addition (*F*_1,74_ = 12.2, *p* = 0.0008), but was not affected by their interaction (*F*_1,74_ = 1.6, *p* = 0.21). Backswimmer presence reduced the proportion of daphniids from an average of 0.067 ± 0.010 to 0.048 ± 0.006 at 10°C, and from an average of 0.15 ± 0.020 to 0.079 ± 0.015 at 18°C (zooplankton samples from Lake Whare had a mean proportion of 0.085 ± 0.05 daphniids; Figure 3b).

Phytoplankton chl *a* was positively affected by temperature (*F*_1,74_ = 4.6, *p* = 0.036) and by backswimmer addition (*F*_1,74_ = 4.3, *p* = 0.043), but there was no significant interaction (*F*_1,74_ = 0.51, *p* = 0.48). Backswimmer presence increased mean phytoplankton chl *a* from 1.9 ± 0.29 to 2.5 ± 0.23 μg/L at 10°C, and from 2.3 ± 0.29 to 3.6 ± 0.47 μg/L at 18°C (Figure 3c).

**Figure 4:**
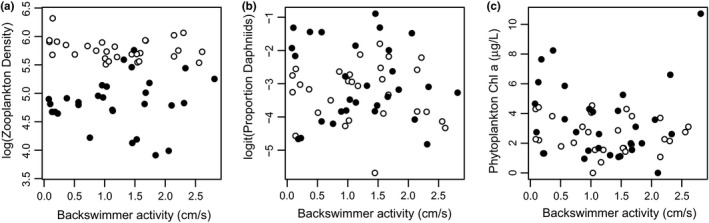
No relationship between *A. assimilis* behavioral type and the strength of top‐down control of (a) zooplankton density, (b) proportion of daphniid zooplankton, and (c) phytoplankton chl *a*. White symbols indicate microcosms in the 10°C treatment and black symbols indicate the 18°C treatment

Backswimmer activity level had no effect on zooplankton density, zooplankton composition, or phytoplankton chl *a* (Figure 4). For zooplankton density, there was a strong effect of temperature (β = −0.93, *p* < 0.0001), but no effect of activity (β = −0.08, *p* = 0.55) or their interaction (β = 0.15, *p* = 0.41; Figure 4a). Sex had no effect (β = −0.05, *p* = 0.61), while backswimmer total length had a marginally significant positive effect on zooplankton density (β = 0.18, *p* = 0.09). Within the microcosms with backswimmers present, the proportion of daphniids was not significantly affected by any predictors (all *p* > 0.1; Figure 4b), and phytoplankton chl *a* was only marginally significantly affected by temperature (β = 0.00097, *p* = 0.07; all other predictors *p* > 0.1; Figure 4c).

## 4 DISCUSSION

Our study found that top‐down control by a freshwater invertebrate predator showed some temperature dependence, but was not affected by predator behavioral type or an interaction between behavioral type and temperature.

Analysis of stable isotope data indicated that *A. assimilis* is largely or exclusively zooplanktivorous in the study pond in austral winter and spring. *A. assimilis* and the closely related *A. wakefieldi* can consume other prey types including larger dipteran larvae and pupae, so their diet is likely to vary across sites and seasons depending on prey availability (Hunt, Galatowitsch, & McIntosh, 2017). Isotope values also showed that there are no obvious relationships between sex or size and diet. We then detected top‐down control in our microcosm setup, confirming that notonectid predators are capable of controlling zooplankton communities (Arner et al., 1998; Blaustein, 1998; Hampton, Gilbert, & Burns, 2000). In fishless ponds, notonectids can play an important role in regulating lower trophic levels, especially when they feed primarily on zooplankton that do not reach a size refuge from predation (Anderson, Kiesecker, Chivers, & Blaustein, 2001). Our results also suggest that *A. assimilis* preferentially predates daphniid cladocerans (in this pond *Ceriodaphnia dubia*) over copepods and other available taxa, as also indicated in experimental studies of *Notonecta* (Arner et al., 1998), and in feeding trials of another New Zealand backswimmer, *A. wakefieldi* (Gilbert & Burns, 1999). Preferential feeding on the more herbivorous *C. dubia* over more omnivorous copepods likely enhances the trophic cascade that manifested in our experiment as a modest yet significant increase in phytoplankton productivity in the presence of a backswimmer.

Our results corroborate a growing body of research indicating that temperature can influence food web processes, and often strengthens top‐down control (Barton et al., 2009; O'Connor et al., 2009; Shurin et al., 2012). At the time of year we carried out our experiment, the elevated temperature treatment can be interpreted as simulating a prolonged heat wave with a daily maximum temperature at the highest end of the range observed in the pond, although we note that our study pond may not be representative of other fishless ponds in the region, and that ponds will experience higher temperatures in the austral summer. As such periods of elevated temperature become more common in the future, we can predict that top‐down control of zooplankton in fishless New Zealand ponds will be strengthened. However, as warming also led to higher relative abundance of *C. dubia*, the effect of temperature in decreasing overall zooplankton density in the presence of backswimmers may be mitigated when it comes to a cascade to primary producers. This may account for the lack of an interactive effect of backswimmer presence and temperature on phytoplankton chl *a*, although we did find that the microcosms with the highest phytoplankton productivities tended to be in the warming + backswimmer treatment. Temperature change can influence predation rates of backswimmers on other prey types including mosquito larvae (Hunt et al., 2017), so temperature‐dependent top‐down control may have broadly important consequences in this type of environment.

We did not detect any effects of predator behavioral type (activity level) or its interaction with temperature on any measure of top‐down control. It is possible that this trait is not related to variation in foraging rate or selectivity in *A. assimilis*, although there are some methodological reasons we may not have detected a true relationship. First, we were only able to assay each backswimmer used in the experiment once. Activity level shows repeatability typical of the range of values reported for behavioral traits across a wide range of taxa (Bell et al., 2009). However, the decline in repeatability when we assayed the same individuals at both temperatures, and the superior fit of a random slopes model, indicate that individuals have heterogeneous reaction norms and activity does not respond consistently to increased temperature (Dingemanse & Dochtermann, 2013; Dingemanse, Kazem, Réale, & Wright, 2010; Kluen & Brommer, 2013; Martin, Nussey, Wilson, & Réale, 2011; van de Pol, 2012). This heterogeneity may reflect true variation among individuals’ thermal performance curves, which would mean that the same temperature change could lead to individuals either increasing or decreasing in activity. However, due to the need to both provide acclimation time to new temperatures and to collect behavioral data soon after collection, the repeated assays at the same temperature were carried out on a shorter timescale (same day) than repeated assays at different temperatures (2 days apart). Over short timescales behavioral repeatability may be overestimated if individuals’ physiological states change over time (Bell et al., 2009; Wexler, Subach, Pruitt, & Scharf, 2016), or may be underestimated if individual differences do not manifest until after a longer acclimation period (Biro, 2012). If individual differences are not consistent across temperatures, we would still expect any effect of activity to manifest at the temperature (10°C) at which experimental backswimmers were assayed. However, if individual repeatability in this behavioral trait erodes over time, individual consistency may not be sufficient to detect effects on food web processes. Ideally, future tests of the effects of individual behavior type on ecosystems should measure repeatability over longer time scales and quantify behavior both before and after, or perhaps during, the experimental period.

Our study is subject to the usual caveats associated with microcosms; in particular, the small spatial scale and simple environment likely increased encounter rates. Zooplankton in fishless ponds are suspected to use vertical migration to reduce predation by invertebrates including notonectids (Gilbert & Hampton, 2001), so the lack of a refuge may have exaggerated the strength of top‐down control in microcosms compared to the pond. While we attempted to keep water levels consistent, it is also possible that greater evaporation in warm microcosms contributed to the enhanced top‐down control by increasing encounter rates. The zooplankton density in the microcosms was also rather higher than in samples collected in the pond, further increasing expected encounter rates. The relatively short duration of the study also meant that our measures of top‐down control primarily captured changes in standing stocks of zooplankton and phytoplankton rather than allowing population dynamics to play out over multiple generations. The microcosms lack other species present in the pond, particularly *A. colensonis* and *S. arguta*, as well as the structural heterogeneity provided by live and dead vegetation. Finally, the availability of only two temperature‐controlled rooms increases the chances that observed temperature effects could be spurious, although the controlled environment and the alternation of which treatment was in which room helps to increase confidence in the results.

A wealth of theory, observational and experimental data indicates that climate warming can have considerable impacts on food web structure and function. At the same time, a growing awareness of the importance of intraspecific variation for ecological processes has highlighted the impact of diversity below the species level (Des Roches et al., 2018). It is therefore natural to ask whether the impacts of trait variation, the expression of which may be temperature‐dependent, will increase or decrease in importance in a warmer world. This question has rarely been asked. A number of studies have measured how temperature impacts both the mean and variance in behavioral traits in populations (Biro et al., 2010; Nakayama et al., 2016; Pruitt et al., 2011), while at least one study has shown that warming enhances the difference in ecological impacts between two divergent populations (Fryxell & Palkovacs, 2017). Additional studies in other systems will be needed to confirm whether intra‐population behavioral variation interacts with temperature to affect food web processes, and traits such as activity are a natural focus for future tests of how individual variation and warming interact.

## CONFLICT OF INTEREST

None declared.

## AUTHORS’ CONTRIBUTIONS

TI conceived the study, TI and ZDB designed the experiment, ZDB carried out the research, TI analyzed the data, TI wrote the manuscript with contributions from ZDB, and all authors approved the final manuscript.

## DATA ACCESSIBILITY

Data available from the Dryad Digital Repository: https://doi.org/10.5061/dryad.b1j16nv.
